# Crystal structure and Hirshfeld surface analysis of (*Z*)-2-{[(2,4-di­methyl­phen­yl)imino]­meth­yl}-4-methyl­phenol

**DOI:** 10.1107/S2056989021010215

**Published:** 2021-10-13

**Authors:** Sevgi Kansiz, Adem Gul, Necmi Dege, Erbil Agar, Eiad Saif

**Affiliations:** a Samsun University, Faculty of Engineering, Department of Fundamental Sciences, 55420, Samsun, Turkey; b Akay Pharma Medicine and Health Products Industries & Trade, 34000, Istanbul, Turkey; c Ondokuz Mayıs University, Faculty of Arts and Sciences, Department of Physics, 55139, Samsun, Turkey; d Ondokuz Mayıs University, Faculty of Arts and Sciences, Department of Chemistry, 55139, Samsun, Turkey; e Department of Computer and Electronic Engineering Technology, Sanaa Community College, Sanaa, Yemen; f Department of Electrical and Electronic Engineering, Faculty of Engineering, Ondokuz Mayıs University, 55139, Samsun, Turkey

**Keywords:** crystal structure, Schiff base, enol–imine tautomer, C—H⋯π inter­actions, Hirshfeld surface analysis

## Abstract

The title compound, C_16_H_17_NO, is a Schiff base that exists in the enol–imine tautomeric form and adopts a *Z* configuration. The mol­ecule is non-planar, with the twisted rings making a dihedral angle of 39.92 (4)°. The intra­molecular O—H⋯N hydrogen bond forms an *S*(6) ring motif. In the crystal, mol­ecules are linked by C—H⋯π inter­actions and very weak π-π stacking inter­actions also help to consolidate the crystal packing. A Hirshfeld surface analysis was performed to investigate the contributions of different inter­molecular contacts within the supra­molecular structure. The major contributions are from H⋯H (65%), C⋯H (19.2%) and O⋯H (6.6%) inter­actions.

## Abstract 2

The title Schiff base exists in the enol–imine tautomeric form and adopts a *Z* configuration. In the crystal, the mol­ecules are linked by weak C—H⋯π hydrogen bonds and very weak π–π stacking inter­actions.

## Chemical context

Schiff bases are well-known organic compounds widely used in many areas. These compounds can be easily synthesized by condensation of a primary aliphatic or aromatic amine with an aldehyde or ketone in different solvent media and they can easily be purified, since the amount of by-products is negligible (Tanak *et al.*, 2020 ▸). Schiff bases are in general more stable than the compounds from which they are synthesized (Wadher *et al.*, 2009 ▸). Nowadays, the possibility of mol­ecular design is an important key for many research areas such as medicine or agriculture. In this respect, Schiff base formation provides an easy way to design new compounds, and biolog­ically or chemically active compounds can be obtained using this method. As the structures of Schiff bases are generally similar to those of biological mol­ecules, Schiff bases are valuable for understanding biological phenomena. As a result, Schiff bases are used in many studies. Various types of aldehydes or ketones have been used for their synthesis, but 2-hy­droxy­benzaldehyde and its derivatives are used especially often (Jeewoth *et al.*, 2000 ▸; Mazhar *et al.*, 2020 ▸). The basis of such preference is the tautomerism and stability provided by the hydroxyl group in conjunction with the imine group. Schiff bases with intra­molecular hydrogen bonds can exhibit photochromic and thermochromic properties (Elerman *et al.*, 2002 ▸). Schiff bases obtained from 2-hy­droxy­benzaldehyde and its derivatives can also form complexes with various metal ions. The title compound is a Schiff base prepared from 2-hy­droxy-5-methyl­benzaldehyde. 
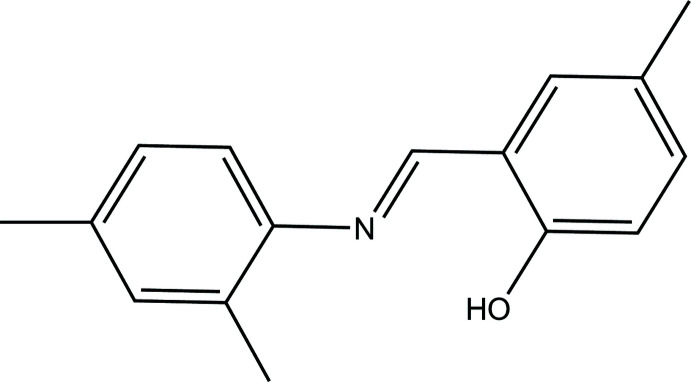


## Structural commentary

**Figure 1:**
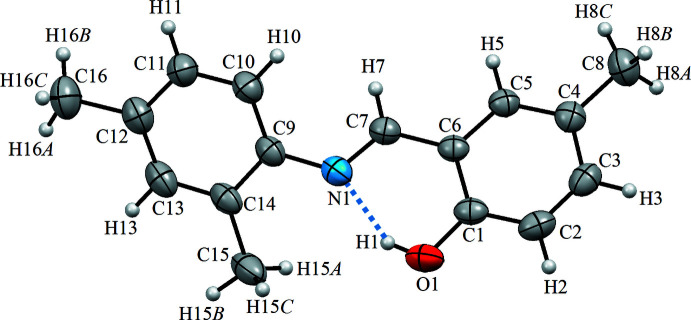
The mol­ecular structure of the title compound, with atom labelling. Displacement ellipsoids are drawn at the 40% probability level. Dashed lines denote the intra­molecular O—H⋯N hydrogen bond forming an *S*(6) ring motif.

The title compound crystallizes in the phenol–imine tautomeric form with an *Z* configuration with respect to the imine bond. The asymmetric unit contains one mol­ecule (Fig. 1 ▸), which is non-planar, two aromatic rings being twisted with respect to each other, subtending a dihedral angle of 39.92 (4)°. The hy­droxy and imine groups are involved in a strong intra­molecular O1—H1⋯N1 hydrogen bond forming an *S*(6) ring motif. The C1—O1 [1.353 (2) Å] and C7—N1 [1.282 (2) Å] bond distances indicate their single- and double-bond characters, respectively, being consistent with the phenol–imine tautomeric form.

## Supra­molecular features

**Figure 2:**
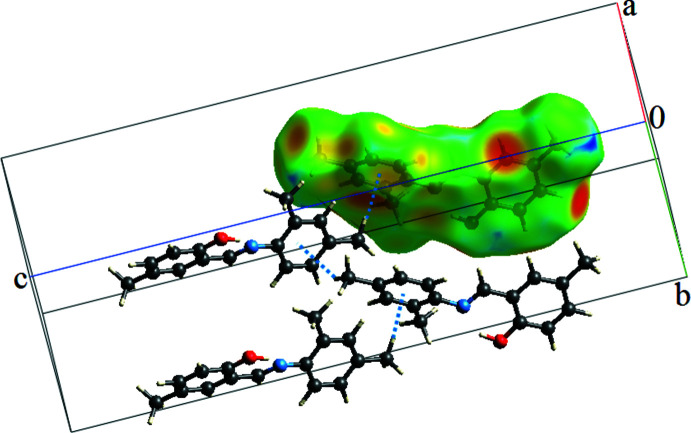
A view of the crystal packing of the title compound. The C16—H16*A*⋯*Cg*2 inter­actions are denoted as dashed lines and as a red spot on the *d_e_* surface.

In the crystal, mol­ecules are linked by C16—H16*A*⋯π (C9–C14) inter­actions (Table 1 ▸, Fig. 2 ▸), and very weak π–π stacking inter­actions between the OH-substituted rings (C1–C6) related by the *a* glide plane [*Cg*⋯*Cg* (− 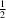 + *x*, *y*, 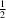 − *z*) = 4.0220 (9) Å] lead to additional stabilization of the crystal packing. A view of the crystal packing parallel to the *bc* plane is shown in Fig. 2 ▸.

## Database survey

A search of the Cambridge Structural Database (CSD, version 5.42, update of May 2021; Groom *et al.*, 2016 ▸) for the (*Z*)-2-{[(2,4-di­methyl­phen­yl)imino]­meth­yl}-4-methyl­phenol unit, revealed ten hits where this fragment adopts the enol–imine tautomeric form. The imine bond length (N1—C7) in the title compound is the same within standard uncertainties as the corresponding bond lengths in the structures of 2-(di­phenyl­meth­yl)-6-[(mesityl­imino)­meth­yl]-4-methyl­phenol (DEHQIS; Zhou *et al.*, 2012 ▸), (*R*)-*N*,*N*′-bis­(3,5-di-*t*-butyl­salicyl­idene)-5,5′,6,6′,7,7′,8,8′-octa­hydro-1,1′-binaphthyl-2,2′-di­amine (MIFXAA; Jia *et al.*, 2002 ▸), aceto­nitrile-bis­{2-(mesitylcarbonoimido­yl)-6-[(mesityl­imino)­meth­yl]-4-methyl­phenolato}magnesium aceto­nitrile solvate (QUDZAS; Ghosh *et al.*, 2015 ▸), bis­{2,4-di-*t*-butyl-6-[(mesityl­imino)­meth­yl]phen­o­lato}tetra­hydro­furan­magnesium (QUDZIA; Ghosh *et al.*, 2015 ▸) and 2,4-di-*t*-butyl-6-{[(2,4,6-tri-*t*-butyl­phen­yl)imino]­meth­yl}phenol (YADZOV; Ma *et al.*, 2016 ▸). As for the C1—O1 bond [1.353 (2) Å], its length compares well with 1.352 (2) Å for YADZOV and 1.359 (5) Å for DEHQIS. All other bond dimensions in the title structure agree well with those in previous literature reports. In NUGWES, NUGWIW and NUGWOC (Xu *et al.*, 2009 ▸) and in YADZOV (Ma *et al.*, 2016 ▸), the lengths of intra­molecular O—H⋯N hydrogen bonds are especially short, being within the range 1.81–1.88 Å.

## Hirshfeld surface analysis

**Figure 4:**
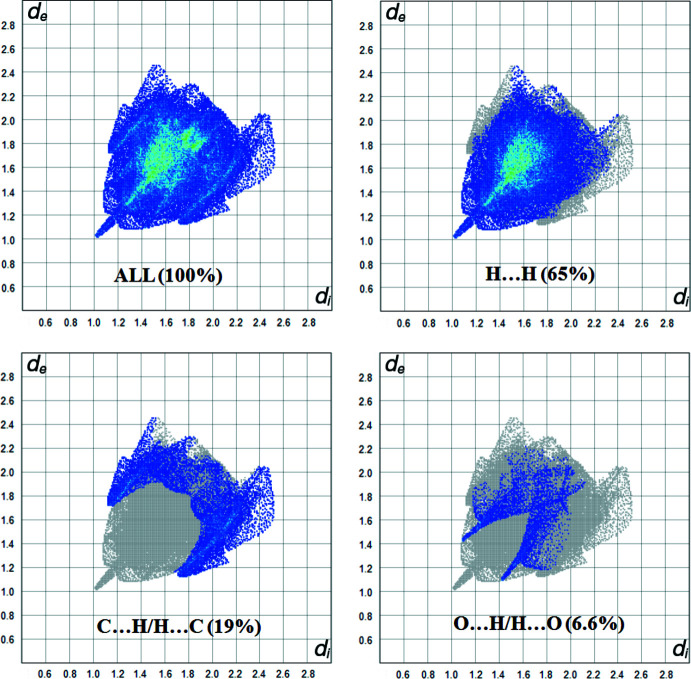
Fingerprint plots showing all inter­molecular inter­actions and resolved into H⋯H, C⋯H/H⋯C and O⋯H/H⋯O contacts.

We have performed a Hirshfeld surface analysis and generated the associated two-dimensional fingerprint plots (Spackman & Jayatilaka, 2009 ▸) with *CrystalExplorer17* (Turner *et al.*, 2017 ▸). Hirshfeld surface analysis is an important way of determining the location of atoms with potential to form hydrogen bonds and other inter­molecular contacts, and the qu­anti­tative ratio of these inter­actions (Demircioğlu *et al.*, 2019 ▸). The Hirshfeld surface was generated using a standard (high) surface resolution with the three-dimensional *d_norm_* surface mapped over a fixed colour scale of −0.1168 (red) to 1.1632 Å (blue) (the fixed colour scale is 1.0201 to 2.4894 Å for the *d_e_* surface). In Figs. 2 ▸ and 3 ▸, the red spots on the *d_norm_* and *d_e_* surfaces represent the C—H⋯*Cg* inter­actions. The most important inter­action is H⋯H, contributing 65% to the overall crystal packing, which is illustrated in the 2D fingerprint (Fig. 4 ▸). Two symmetrical wings on the left and right sides are seen in the fingerprint plot for C⋯H/H⋯C inter­actions, the second most important contributor to the total Hirshfeld surface (19%). The O⋯H/H⋯O inter­actions provide a 6.6% contribution to the total Hirshfeld surface. Much weaker C⋯C (5.3%), N⋯H/H⋯N (2.3%) and C⋯O/O⋯C (1.3%) contacts are also present.

## Synthesis and crystallization

**Figure 5:**
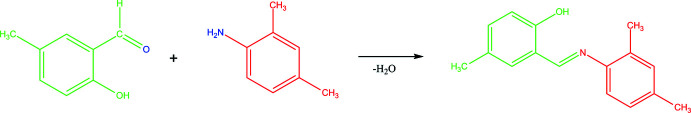
The scheme of synthesis of the title compound.

(*Z*)-2-{[(2,4-di­methyl­phen­yl)imino]­meth­yl}-4-methyl­phenol was synthesized by condensation of 2-hy­droxy-5-methyl­benzaldehyde and 2,4-di­methyl­aniline (Fig. 5 ▸). For this purpose, a mixture of a solution containing 2-hy­droxy-5-methyl­benzaldehyde (0.04 mmol) in ethanol (20 mL) and a solution containing 2,4-di­methyl­aniline (0.04 mmol) in ethanol (20 mL) was refluxed for 6 h under stirring. The obtained crystalline product was washed with ethanol and dried at room temperature. Single crystals were obtained by slow evaporation of ethanol solution at room temperature.

## Refinement

Crystal data, data collection and structure refinement details are summarized in Table 2 ▸. The O-bound H atom was located in a difference-Fourier map and refined with O—H = 0.82 Å, and with *U*_iso_(H) = 1.5*U*_eq_(O). The C-bound H atoms were positioned geometrically and refined using a riding model with C—H = 0.93 and *U*_iso_(H) = 1.2*U*_eq_(C) for *sp* ^2^-hybridized C atoms and with C—H = 0.96 Å and *U*_iso_(H) = 1.5*U*_eq_(C) for methyl groups.

## Supplementary Material

Crystal structure: contains datablock(s) I. DOI: 10.1107/S2056989021010215/yk2157sup1.cif

Structure factors: contains datablock(s) I. DOI: 10.1107/S2056989021010215/yk2157Isup2.hkl

Supporting information file. DOI: 10.1107/S2056989021010215/yk2157Isup3.cml

CCDC reference: 2113562

Additional supporting information:  crystallographic information; 3D view; checkCIF report

## Figures and Tables

**Figure 3:**
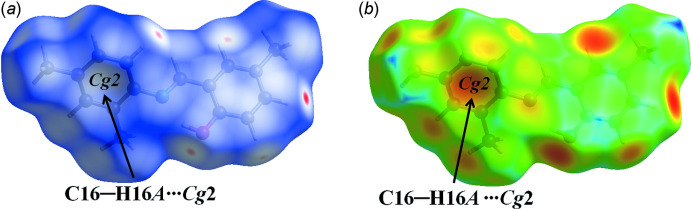
The red spots on the *d_norm_ and d_e_* surfaces of the title mol­ecule represent the C—H⋯π inter­actions.

**Table 1:** Hydrogen-bond geometry (Å, °) *Cg*2 is the centroid of the C9–C14 ring.

*D*—H⋯*A*	*D*—H	H⋯*A*	*D*⋯*A*	*D*—H⋯*A*
O1—H1⋯N1	0.82	1.89	2.618 (2)	147
C16—H16*A*⋯*Cg*2^i^	0.96	2.93 (3)	3.73	143

Symmetry code: (i) x+{\script{1\over 2}}, -y+{\script{1\over 2}}, -z.

**Table 2:** Experimental details

Crystal data	
Chemical formula	C_16_H_17_NO
*M*_r_	239.30
Crystal system, space group	Orthorhombic, *P* *b* *c* *a*
Temperature (K)	296
*a*, *b*, *c* (Å)	7.6699 (4), 11.6080 (6), 30.1431 (17)
*V* (Å^3^)	2683.7 (2)
*Z*	8
Radiation type	Mo *K*α
μ (mm^−1^)	0.07
Crystal size (mm)	0.68 × 0.48 × 0.18

Data collection	
Diffractometer	Stoe IPDS 2
Absorption correction	Integration (*X-RED32*; Stoe & Cie, 2002 ▸)
*T*_min_, *T*_max_	0.953, 0.990
No. of measured, independent and observed [*I* > 2σ(*I*)] reflections	14869, 2213, 1451
*R*_int_	0.081
(sin θ/λ)_max_ (Å^−1^)	0.583

Refinement	
*R*[*F* ^2^ > 2σ(*F* ^2^)], *wR*(*F* ^2^), *S*	0.042, 0.125, 1.00
No. of reflections	2213
No. of parameters	168
H-atom treatment	H-atom parameters constrained
Δρ_max_, Δρ_min_ (e Å^−3^)	0.11, −0.11

Computer programs: *X-AREA* (Stoe & Cie, 2002 ▸), *X-RED* (Stoe & Cie, 2002 ▸), *SHELXT2017/1* (Sheldrick, 2015*a* ▸), *SHELXL2017/1* (Sheldrick, 2015*b* ▸), *PLATON* (Spek, 2020 ▸), *WinGX* (Farrugia, 2012 ▸).

## supplementary crystallographic information

### Crystal data

**Table:**

C_16_H_17_NO	*D*_x_ = 1.185 Mg m^−^^3^
*M**_r_* = 239.30	Mo *K*α radiation, λ = 0.71073 Å
Orthorhombic, *P**b**c**a*	Cell parameters from 13620 reflections
*a* = 7.6699 (4) Å	θ = 1.4–24.9°
*b* = 11.6080 (6) Å	µ = 0.07 mm^−^^1^
*c* = 30.1431 (17) Å	*T* = 296 K
*V* = 2683.7 (2) Å^3^	Plate, orange
*Z* = 8	0.68 × 0.48 × 0.18 mm
*F*(000) = 1024	

### Data collection

**Table:**

Stoe IPDS 2 diffractometer	2213 independent reflections
Radiation source: sealed X-ray tube, 12 x 0.4 mm long-fine focus	1451 reflections with *I* > 2σ(*I*)
Detector resolution: 6.67 pixels mm^-1^	*R*_int_ = 0.081
rotation method scans	θ_max_ = 24.5°, θ_min_ = 1.4°
Absorption correction: integration (X-RED32; Stoe & Cie, 2002)	*h* = −8→8
*T*_min_ = 0.953, *T*_max_ = 0.990	*k* = −13→13
14869 measured reflections	*l* = −34→34

### Refinement

**Table:**

Refinement on *F*^2^	Hydrogen site location: inferred from neighbouring sites
Least-squares matrix: full	H-atom parameters constrained
*R*[*F*^2^ > 2σ(*F*^2^)] = 0.042	*w* = 1/[σ^2^(*F*_o_^2^) + (0.0725*P*)^2^] where *P* = (*F*_o_^2^ + 2*F*_c_^2^)/3
*wR*(*F*^2^) = 0.125	(Δ/σ)_max_ < 0.001
*S* = 1.00	Δρ_max_ = 0.11 e Å^−^^3^
2213 reflections	Δρ_min_ = −0.11 e Å^−^^3^
168 parameters	Extinction correction: file:///iucrfs/e/yk2157/yk2157.cif, Fc^*^=kFc[1+0.001xFc^2^λ^3^/sin(2θ)]^-1/4^
0 restraints	Extinction coefficient: 0.0094 (14)
Primary atom site location: structure-invariant direct methods	

### Special details

**Table:**

Geometry. All esds (except the esd in the dihedral angle between two l.s. planes) are estimated using the full covariance matrix. The cell esds are taken into account individually in the estimation of esds in distances, angles and torsion angles; correlations between esds in cell parameters are only used when they are defined by crystal symmetry. An approximate (isotropic) treatment of cell esds is used for estimating esds involving l.s. planes.

### Fractional atomic coordinates and isotropic or equivalent isotropic displacement parameters (Å^2^)

**Table:**

	*x*	*y*	*z*	*U*_iso_*/*U*_eq_	
O1	0.3099 (2)	0.58146 (10)	0.30727 (4)	0.0844 (4)	
H1	0.330751	0.540427	0.328769	0.127*	
N1	0.42492 (18)	0.40012 (12)	0.34984 (5)	0.0663 (4)	
C6	0.4130 (2)	0.40987 (13)	0.27056 (6)	0.0569 (4)	
C5	0.4477 (2)	0.35487 (14)	0.23014 (5)	0.0595 (4)	
H5	0.495649	0.281288	0.230684	0.071*	
C7	0.4516 (2)	0.35261 (15)	0.31204 (6)	0.0611 (5)	
H7	0.497415	0.278473	0.311267	0.073*	
C1	0.3426 (2)	0.52223 (13)	0.26953 (6)	0.0630 (5)	
C9	0.4629 (2)	0.33967 (15)	0.38970 (6)	0.0633 (5)	
C4	0.4139 (2)	0.40502 (16)	0.18961 (6)	0.0648 (5)	
C14	0.5297 (2)	0.40228 (16)	0.42555 (6)	0.0692 (5)	
C10	0.4302 (2)	0.22231 (16)	0.39438 (6)	0.0724 (5)	
H10	0.383752	0.180914	0.370760	0.087*	
C2	0.3077 (2)	0.57301 (15)	0.22917 (7)	0.0739 (5)	
H2	0.259857	0.646596	0.228269	0.089*	
C3	0.3428 (2)	0.51607 (16)	0.19032 (7)	0.0736 (5)	
H3	0.318475	0.552460	0.163539	0.088*	
C12	0.5363 (2)	0.22599 (19)	0.46974 (6)	0.0759 (6)	
C13	0.5668 (2)	0.34303 (19)	0.46426 (6)	0.0771 (6)	
H13	0.614731	0.383787	0.487863	0.093*	
C11	0.4663 (3)	0.16695 (17)	0.43391 (6)	0.0774 (5)	
H11	0.443232	0.088594	0.436544	0.093*	
C8	0.4528 (3)	0.34410 (19)	0.14659 (6)	0.0893 (6)	
H8A	0.524423	0.392488	0.128285	0.134*	
H8B	0.345600	0.327600	0.131447	0.134*	
H8C	0.513250	0.273397	0.152645	0.134*	
C15	0.5618 (3)	0.53050 (18)	0.42172 (7)	0.0970 (7)	
H15A	0.638745	0.545186	0.397253	0.145*	
H15B	0.613945	0.558242	0.448631	0.145*	
H15C	0.453020	0.569432	0.416857	0.145*	
C16	0.5729 (3)	0.1666 (2)	0.51352 (7)	0.1014 (7)	
H16A	0.650363	0.213308	0.530910	0.152*	
H16B	0.625932	0.093075	0.508055	0.152*	
H16C	0.465487	0.155857	0.529352	0.152*	

### Atomic displacement parameters (Å^2^)

**Table:**

	*U*^11^	*U*^22^	*U*^33^	*U*^12^	*U*^13^	*U*^23^
O1	0.0935 (10)	0.0608 (7)	0.0990 (10)	0.0078 (7)	0.0082 (9)	−0.0177 (7)
N1	0.0658 (9)	0.0681 (9)	0.0649 (10)	−0.0063 (7)	0.0056 (8)	−0.0106 (8)
C6	0.0514 (9)	0.0516 (9)	0.0676 (10)	−0.0051 (7)	−0.0008 (8)	−0.0042 (8)
C5	0.0546 (9)	0.0567 (9)	0.0673 (11)	−0.0019 (7)	−0.0042 (9)	−0.0018 (9)
C7	0.0588 (10)	0.0572 (10)	0.0672 (11)	−0.0014 (8)	0.0001 (9)	−0.0053 (9)
C1	0.0544 (9)	0.0532 (9)	0.0813 (12)	−0.0049 (7)	0.0020 (10)	−0.0063 (10)
C9	0.0585 (10)	0.0731 (12)	0.0582 (10)	−0.0035 (8)	0.0057 (9)	−0.0098 (9)
C4	0.0552 (10)	0.0704 (12)	0.0686 (12)	−0.0072 (8)	−0.0058 (9)	0.0003 (9)
C14	0.0639 (11)	0.0820 (12)	0.0618 (11)	−0.0096 (9)	0.0138 (9)	−0.0186 (10)
C10	0.0748 (12)	0.0757 (12)	0.0667 (12)	−0.0056 (9)	−0.0078 (10)	−0.0093 (9)
C2	0.0638 (11)	0.0548 (10)	0.1032 (15)	−0.0006 (8)	−0.0073 (11)	0.0066 (11)
C3	0.0672 (12)	0.0713 (12)	0.0822 (14)	−0.0090 (10)	−0.0129 (10)	0.0163 (10)
C12	0.0647 (11)	0.0989 (15)	0.0642 (12)	0.0014 (10)	−0.0002 (9)	−0.0072 (10)
C13	0.0672 (12)	0.1049 (16)	0.0593 (11)	−0.0127 (11)	0.0051 (9)	−0.0186 (10)
C11	0.0842 (13)	0.0762 (12)	0.0716 (12)	0.0001 (10)	−0.0075 (11)	−0.0006 (10)
C8	0.0923 (15)	0.1077 (16)	0.0680 (13)	0.0019 (12)	−0.0081 (11)	−0.0041 (11)
C15	0.1220 (19)	0.0904 (15)	0.0785 (13)	−0.0314 (13)	0.0196 (13)	−0.0273 (11)
C16	0.1019 (17)	0.130 (2)	0.0727 (14)	−0.0012 (14)	−0.0154 (12)	0.0056 (13)

### Geometric parameters (Å, º)

**Table:**

O1—C1	1.353 (2)	C10—H10	0.9300
O1—H1	0.8200	C2—C3	1.371 (3)
N1—C7	1.2823 (19)	C2—H2	0.9300
N1—C9	1.421 (2)	C3—H3	0.9300
C6—C5	1.401 (2)	C12—C11	1.387 (3)
C6—C1	1.412 (2)	C12—C13	1.388 (3)
C6—C7	1.447 (2)	C12—C16	1.515 (3)
C5—C4	1.378 (2)	C13—H13	0.9300
C5—H5	0.9300	C11—H11	0.9300
C7—H7	0.9300	C8—H8A	0.9600
C1—C2	1.378 (2)	C8—H8B	0.9600
C9—C10	1.392 (2)	C8—H8C	0.9600
C9—C14	1.400 (2)	C15—H15A	0.9600
C4—C3	1.400 (3)	C15—H15B	0.9600
C4—C8	1.507 (3)	C15—H15C	0.9600
C14—C13	1.384 (3)	C16—H16A	0.9600
C14—C15	1.513 (3)	C16—H16B	0.9600
C10—C11	1.382 (2)	C16—H16C	0.9600
			
C1—O1—H1	109.5	C2—C3—H3	118.9
C7—N1—C9	120.40 (15)	C4—C3—H3	118.9
C5—C6—C1	118.32 (16)	C11—C12—C13	117.14 (18)
C5—C6—C7	120.23 (15)	C11—C12—C16	121.7 (2)
C1—C6—C7	121.44 (16)	C13—C12—C16	121.16 (18)
C4—C5—C6	122.88 (16)	C14—C13—C12	123.48 (17)
C4—C5—H5	118.6	C14—C13—H13	118.3
C6—C5—H5	118.6	C12—C13—H13	118.3
N1—C7—C6	122.54 (16)	C10—C11—C12	121.27 (19)
N1—C7—H7	118.7	C10—C11—H11	119.4
C6—C7—H7	118.7	C12—C11—H11	119.4
O1—C1—C2	119.28 (16)	C4—C8—H8A	109.5
O1—C1—C6	121.45 (17)	C4—C8—H8B	109.5
C2—C1—C6	119.26 (17)	H8A—C8—H8B	109.5
C10—C9—C14	119.71 (17)	C4—C8—H8C	109.5
C10—C9—N1	122.14 (15)	H8A—C8—H8C	109.5
C14—C9—N1	118.12 (16)	H8B—C8—H8C	109.5
C5—C4—C3	116.66 (17)	C14—C15—H15A	109.5
C5—C4—C8	121.83 (17)	C14—C15—H15B	109.5
C3—C4—C8	121.50 (17)	H15A—C15—H15B	109.5
C13—C14—C9	117.93 (17)	C14—C15—H15C	109.5
C13—C14—C15	121.31 (17)	H15A—C15—H15C	109.5
C9—C14—C15	120.76 (18)	H15B—C15—H15C	109.5
C11—C10—C9	120.43 (17)	C12—C16—H16A	109.5
C11—C10—H10	119.8	C12—C16—H16B	109.5
C9—C10—H10	119.8	H16A—C16—H16B	109.5
C3—C2—C1	120.63 (17)	C12—C16—H16C	109.5
C3—C2—H2	119.7	H16A—C16—H16C	109.5
C1—C2—H2	119.7	H16B—C16—H16C	109.5
C2—C3—C4	122.24 (17)		
			
C1—C6—C5—C4	−0.9 (2)	N1—C9—C14—C15	−0.5 (3)
C7—C6—C5—C4	−179.97 (15)	C14—C9—C10—C11	−1.2 (3)
C9—N1—C7—C6	179.20 (14)	N1—C9—C10—C11	−178.95 (16)
C5—C6—C7—N1	178.78 (14)	O1—C1—C2—C3	178.64 (16)
C1—C6—C7—N1	−0.3 (2)	C6—C1—C2—C3	−0.8 (3)
C5—C6—C1—O1	−178.36 (15)	C1—C2—C3—C4	0.3 (3)
C7—C6—C1—O1	0.7 (2)	C5—C4—C3—C2	−0.1 (3)
C5—C6—C1—C2	1.1 (2)	C8—C4—C3—C2	−179.56 (17)
C7—C6—C1—C2	−179.83 (15)	C9—C14—C13—C12	−2.0 (3)
C7—N1—C9—C10	−38.6 (2)	C15—C14—C13—C12	178.60 (18)
C7—N1—C9—C14	143.67 (16)	C11—C12—C13—C14	0.6 (3)
C6—C5—C4—C3	0.4 (2)	C16—C12—C13—C14	−177.52 (18)
C6—C5—C4—C8	179.87 (15)	C9—C10—C11—C12	−0.3 (3)
C10—C9—C14—C13	2.3 (3)	C13—C12—C11—C10	0.6 (3)
N1—C9—C14—C13	−179.87 (15)	C16—C12—C11—C10	178.71 (19)
C10—C9—C14—C15	−178.33 (17)		

### Hydrogen-bond geometry (Å, º)

*Cg*2 is the centroid of the C9–C14 ring.

**Table:**

*D*—H···*A*	*D*—H	H···*A*	*D*···*A*	*D*—H···*A*
O1—H1···N1	0.82	1.89	2.618 (2)	147
C16—H16*A*···*Cg*2^i^	0.96	2.93 (3)	3.73	143

Symmetry code: (i) *x*+1/2, −*y*+1/2, −*z*.
